# Monetary Value of Quality-Adjusted Life Years (QALY) among Patients with Cardiovascular Disease: a Willingness to Pay Study (WTP)

**Published:** 2017

**Authors:** Najmeh Moradi, Arash Rashidian, Hamid Reza Rasekh, Alireza Olyaeemanesh, Mahnoosh Foroughi, Teymoor Mohammadi

**Affiliations:** a *School of Pharmacy ,Shahid Beheshti University of medical sciences, Tehran, Iran. *; b *School of Public Health, Tehran University of Medical Sciences, Tehran, Iran.*; c *School of Pharmacy, Shahid Beheshti University of Medical Sciences, Tehran, Iran. *; d *Health Economics Department, National Institute for Health Research, Tehran University of Medical Sciences, Tehran, Iran. *; e *cardiovascular research center, Shahid Beheshti University of medical sciences, Tehran, Iran. *; f *Economics Faculty, Allameh Tabatabaei University, Tehran Iran.*

**Keywords:** Willingness To Pay, Quality-Adjusted Life Year, Contingent Valuation Method, Cost -Effectiveness threshold value, Cardiovascular diseases

## Abstract

The aim of this study was to estimate the monetary value of a QALY among patients with heart disease and to identify its determinants. A cross-sectional survey was conducted through face-to-face interview on 196 patients with cardiovascular disease from two heart hospitals in Tehran, Iran, to estimate the value of QALY using disaggregated and aggregated approaches. The EuroQol-5 Dimension (EQ-5D) questionnaire, Visual Analogue Scale (VAS), Time Trade-Off (TTO) and contingent valuation WTP techniques were employed, first to elicit patients’ preferences and then, to estimate WTP for QALY. The association of patients’ characteristics with WTP for QALY, was assessed through Heckman selection model. The Mean willingness to pay per QALY, estimated by the disaggregated approach ranged from 2,799 to 3599 US dollars. It is higher than the values, estimated from aggregated methods (USD 2,256 to 3,137). However, in both approaches, the values were less than one Gross Domestic Product (GDP) per capita of Iran.

Significant variables were: Current health state, education, age, marital status, number of comorbidities, and household’s cost group. Our results challenge two major issues: the first, is a policy challenge which concerns the WHO recommendation to use less than 3 GDP per capita as a cost-effectiveness threshold value. The second, is an analytical challenge related to patients with zero QALY gain. More scrutiny is suggested on the issue of how patients with full health state valuation should be dealt with and what arbitrary value could be included in the estimation value of QALY when the disaggregated approach used.

## Introduction

Cardiovascular diseases (CVDs), are one of the most life-threatening diseases in the world because of fatal consequences which are mainly due to the disease itself and related comorbidities (such as diabetes or hypertension). It was the leading cause of death among non-communicable diseases (NCD) in 2008 by the largest proportion of deaths, namely 17 million cases of death among 36 million in NCD and 30% of all deaths that occurred in the world (1). It was also estimated that by 2030, more than 23 million deaths will occur from CVDs annually (2). Additionally, CVDs are one of the leading causes of Disability-Adjusted Life Years (DALY) in the world due to premature death and disabling consequences (3). Apart from negative impacts of CVDs on patients, they also have imposed enormous costs on economy, including treatments costs and costs of productivity loss due to premature death, as well as morbidity and disabling conditions. The study on economic burden of CVDs across six major European economies in 2014 showed that the total costs of diseases, equal GDP of a middle size European economy, like Hungary. It was also indicated that inpatient care and pharmaceutical costs are the major health-care costs of CVDs, respectively (4). In Iran, the ischemic heart disease has the highest rank in the number of Years of Life Lost (YLLs), which was due to the premature deaths in 2010 (5).

Therefore, there is a general consensus in all health systems around the world for reducing negative impacts of diseases on individuals and society by managing and controlling CVDs. In this way, within limited health care resource settings, economic analysis, especially in the form of cost-effectiveness analysis (CEA) is one of the most important tools, by helping decision makers as a guideline in priority setting, resource allocation and reimbursement decisions related to treating and managing CVDs (1, 6). In CEA analysis, different competitive interventions were compared, in terms of their costs and health effects (outcomes) and result is typically expressed as an Incremental Cost-Effectiveness Ratio (ICER), the ratio of differences in costs of alternative interventions to differences in outcomes (7). Among different outcome measures of CEA for reporting ICER, such as the single specific-health effect like, life-years gained or the number of cases found, Quality-Adjusted Life Year (QALY), or averted DALY, QALY is a well-accepted outcome measure that incorporates both improvement in quantity and (or) quality of life in a single index. It is a key input in informed decision making that facilitates the comparison of different interventions within or outside the health system (8). Incremental Cost per QALY indicates the costs which are associated with QALY gain and determines the amount of money that should be allocated to achieve one extra QALY. Therefore, by knowing the monetary value of QALY as a threshold value, it could be a guide to reduce the burden of CVDs through determining interventions, which produce the highest gains for the health system and society and worth buying. This value could be derived from general public or patients’ perspective for general health state or specific health state via survey or modelling approaches for policy implications and reimbursement decisions (9). In recent years, there has been a rapid growth in the number of studies trying to estimate the value of QALY in the form of survey through contingent valuation Willingness To Pay (WTP) method at national and international levels (10-12). At present, this value is not determined for Iran’s health system. Therefore, to interpret the results of CEA analysis, the proposed criteria by World Health Organization (WHO) on 1-3 times of GDP per capita were used as a threshold value (13).

In this pilot study, by considering the overall burden of CVDs on the health system, and also the need to define the evidence–based threshold value, we aimed to determine the value of QALY with these purposes: 1) measuring the monetary value of QALY from patients’ perspective with CVDs through WTP technique, 2) exploring determinant factors affecting this value and 3) discussing health policy implications of the results, especially with examining its consistency of it with WHO recommendations. 

## Methods

*Study design*

The present study is a descriptive and analytical cross-sectional study. A face-to-face interview based on the detailed protocol was conducted with eligible heart patients. This was done to elicit health and WTP preferences.

*Study populations*

Hospitalized patients with cardiovascular diseases who referred from the coronary care unit (CCU) or post-CCU were interviewed by trained interviewers from September 2014 to January 2015. Other eligibility criteria included age, which was determined to be more than 18 years-old with ability to understand and speak in Persian. It should be noted that an informed written consent form was obtained from patients before the interview. The study protocol was approved by the Ethics Committee of Shahid Beheshti University of Medical science (SBMUS).

Our samples were recruited from two heart hospitals, Shahid Rajaei Cardiovascular Medical and Research Center of Iran University of Medical Sciences and Shahid Modarres cardiovascular research center of SBMU as referral hospitals that render professional services to patients with different types of heart diseases. Patients’ characteristics such as socioeconomic, demographic and disease-specific variables were extracted using a questionnaire. Age, sex, marriage status, head of household, education, employment and cost group as a proxy for monthly household’s income, were considered as demographic and socioeconomic variables. Current health status, type of CVDs and the type of related complications or comorbidities such as: Hypertension, high cholesterol, diabetes, respiratory, kidney and eye diseases, and stroke were considered as disease-specific variables. Other factors were: patients’ hospitalization experience in the past year, hospitalization of any household member in the past year, and near-death experience in family in the past year.

*Questionnaire development*

The primary questionnaire to measure and monetary valuation of QALY was designed in 5 sections: introduction, health utility measurement, WTP measurement, and individual characteristics. In the introduction section, we explained the aims of the survey for respondents, the types of questions we asked and also we emphasized on confidentiality of gathered information. The questionnaire was pretested in 15 pilot samples to examine feasibility of the study and to determine WTP distribution. The final questionnaire was modified based on pilot results including use of close-ended payment questions instead of open-ended ones to elicit WTP. In addition, based on the results of the pilot study, supplementary questions were added for patients with zero answers to preference questions.

*Preferences elicitation*

We elicited patients’ preferences from two steps. At first, patients were interviewed to elicit their health utility through common health preference measures, directly through VAS and TTO techniques and indirectly using the Persian-validated EQ-5D, and then, their WTP were elicited.

In VAS, patients were asked to rate their own current health state on a vertical line ranging from 0 as death to 100 as the full health state. The patient’s utility value was calculated by dividing the rated score by 100 (14).

EQ_5D is a multi-attribute generic preference measure, which evaluates patients’ health state based on the five dimensions including: Mobility, self-care, usual activity, pain and discomfort, and anxiety and depression in three levels of ‘no problem’, ‘some problems’, and ‘severe problems’ (15). In this study, the health utility value associated with each health state, was calculated by employing the value set, recently generated for Iranian population (16).

In TTO and WTP techniques, all patients were presented with a hypothetical scenario on a treatment with these features: safe, new, without pain and side effects which recover them to full health definitely and immediately, but for obtaining this treatment, patients should trade time or money. The TTO technique is based on the trade-off between quantity and quality of life. In this method, patients were asked, how much time they would be willing to exchange for a shorter life in full health instead of spending the rest of their life in current health. In current TTO valuation exercise, two time life-spans were used: Adjusted with life expectancy, and fixed 10-years, irrespective of patients’ age. To elicit WTP, patients were made sure that they did not need to trade any life-time, but the treatment was not covered by the government or health insurance and they are supposed to pay for it from their own pocket. Out of pocket payment was chosen as an appropriate payment vehicle as it was recommended in CVM surveys. It is also a common payment form in Iran’ health system and is more realistic payment way for patients (17, 18). Also, it is more realistic payment way for patients  by reason of it’s a common payment form in Iran’s health system. 

*WTP measurement*

To ask the WTP question, we used the Contingent Valuation Method (CVM), a survey- based technique that widely used for the monetary valuation of non-market goods, such as environmental or health (19). CVM is a stated preference model asking people how much money they would be willing to pay (willing to accept), to achieve (foregone) a benefit (20). It was first introduced by S.V. Ciriacy Wantrup at 1947 as a method for eliciting market valuation of a non-market good. It would be interesting to know that for the first time, it was used in health care by Acton in 1973 to estimate WTP for reducing the risk of death from heart attack through improved ambulance services (21). 

**Table 1:** Descriptive statistics of patients ’actual costs and open-ended WTP question

	**Min (non-zero)**	**Max**	**mean**	**median**
Pilot test	500,000	100,000,000	30,000,000	2,500,000
Actual cost	4,222,000	300,000,000	90,000,000	80,000,000

Currency is in Iranian Rials (IRR

**Table 2:** Bid values used in DBDC technique

**Number**	**Initial bid**	**Bid up**	**Bid low**
1 2 3 4 5 6 7 8 9	5000,000 10,000,000 30,000,000 50,000,000 70,000,000 90,000,000 110,000,000 150,000,000 200,000,000	10,000,000 30,000,000 50,000,000 70,000,000 90,000,000 110,000,000 150,000,000 200,000,000 300,000,000	2,500,000 5000,000 10,000,000 30,000,000 50,000,000 70,000,000 90,000,000 110,000,000 150,000,000

The bid values are in IRR

**Table 3:** Characteristics of Patients

**age**	56.6 (54.84,58.36)
male	140 (72%)
Married	159 (82%)
Head of household	151 (78%)
Household size	3.49 (3.24,3.72)
**education**	
illiterate	34 (17.53%)
primary education	67 (34.53)
Secondary education	19 (9.79%)
High school diploma	47 ( 24.23)
University education	27 (13.92%)
**employment**	
Having job or income	119 (65.03%)
**Cost group (1000,000 IRR)**	
< o.5	26 (13.40%)
0.5-1	66 (34.02%)
1-2	68 (35.05%)
2-3	24 (12.37%)
>3	10 (5.16%)
**diagnosis**	
Coronary artery disease	111 (57.22%)
Heart failure	21 (10.82%)
Arrhythmia	15 (7.73%)
**Other diagnosis**	
Hospitalization experience at last year	81 (41.75%)

**Table 4:** Results of patients’ preferences measurement (health and WTP

**preference measure**	**EQ-5D**	**VAS**	**TTO(ADJUST)**	**TTO (10-year)**	**WTP(IRR)(10,000,000)**
Mean ± SD	0.59 ± 0.31	0.62 ± 0.23	0.71 ± 0.22	0.71± 0.25	300 ± 80
Confidence interval (95%)	0.55,0.64	0.59, 0.65	0.68,0.74	0.68,0.75	180, 420
Minimum	0	0	0.07	0.1	0
Maximum	1	1	1	1	8000
no of respondents with zero value (QALY and WTP)	22%(43)*	2%(4)*	21%(40)**	26%(51)**	17% (33)***

* = number of respondents with full health state valuation,

**= number of respondents who unwilling to trade time,

*** = number of respondents who unwilling to pay

**Table 5:** Mean WTP for QALY derived from different preference measures and the ratio of it to GDP per capita

** Preference measures**	**EQ-5D**	**VAS**	**TTO** **)** **Adjusted** **(**	**TTO** **)** **10-years** **(**
**Mean value**				
Disaggregated WTP/QALY±(SD)	48,350,730 ±1.810E+08	64,734,470 ±2.308E+08	55,478,110 ±1.485E+08	58,068,950 ±1.745E+08
discounted WTP/QALY (.05) (SD)	76,168,710 ±2.812E8	100,767,560 ±3.374E8	96,478,230 ±2.903E8	95,970,290 ±2.727E8
The ratio of WTP/QALY to GDP per capita	0.57	0.76	0.72	0.72

Discount rate=R=0.05, currency: Iranian Rials, SD=Standard Deviation

**Table 6:** The relationship between Ln(WTP/QALY) and patients’ characteristics with 0.95 confidence interval

	**VAS**		**EQ-5D**		**TTO-ADJUSTED**		**TTO-10 YEAR**	
dependents variables	Coef. (Std. Err.)	p-value	Coef. (Std. Err.)	p-value	Coef. (Std. Err.)	p-value	Coef. (Std. Err.)	p-value
current health	4.929545 (1.770054)	0.005	4.843574 (1.861025)	0.009	3.735546 (1.987528)	0.060	4.197557 (1.833432)	0.022
age	-.1115419 ).0335865(	0.001	-.1019878 ( .0391688)	0.009	-.0532749 ( .034318)	0.121	-.0556592 (.0360117)	0.122
Edu	.8664595 ).2529248(	0.001	1.018456 (2655811)	0.000	1.197845 (.2351781)	0.000	1.143632 (.2563346)	0.000
no. comorbid	.5343068 ).2233258(	0.017	.7634571 ( .29118)	0.009	.3046931 (.2177222)	0.162	.2390456 (.2287948)	0.296
Marriage	not included		1.365139 ( 1.098373)	0.214	1.722562 (.9699506)	0.076	1.443428 (1.01676)	0.156
Cost group	.7026398 ).392879(	0.074	.8638929 ( .4068036)	0.034	.5437692 (.3708306)	0.143	.6604489 (.3881864)	0.089
Constant	9.471611 (2.429139)	0.000	6.061894 ( 2.946571)	0.040	5.24437 (2.804221)	0.061	5.31931 (2.709118)	0.050
Number of obs	194		194		194		192	
Censored obs	4		43		40		51	
Wald chi2	45.98		56.74		51.09		45.95	
Prob> chi2	0		0		0		0	
Log likelihood	-592.0015		-546.0258		-543.2941		-520.6449	
Athrho	-15.45812 124.8649	0.901	.0847952 .3747231	0.821	.2241023 .3079969	0.467	.191291 .3821206	0.617
Lnsigma	1.674162 .0507048	0.000	1.601962 .0588219	0.000	1.497194 .0637308	0.000	1.520298 .0681676	0.000
Rho	-1 1.87e-11		.0845925 .3720417		.2204245 .2930323		.1889914 .3684721	
Sigma	5.334325 .2704758		4.96276 .291919		4.46913 .284821		4.573586 .3117703	
Lambda	-5.334325 .2704758		.4198124 1.851641		.9851058 1.338976		.8643685 1.714723	
	LR test of indep. eqns. (rho = 0): chi2(1) = 23.51 Prob > chi2 = 0.0000		LR test of indep. eqns. rho = 0): chi2(1) = 0.04 Prob > chi2 = 0.833		LR test of indep. eqns. (rho =0): chi2(1) = .36 Prob > chi2 =0.5500		LR test of indep. eqns. (rho = 0): chi2(1) =0.15 Prob > chi2 = 0.6945	

**Figure 1:**
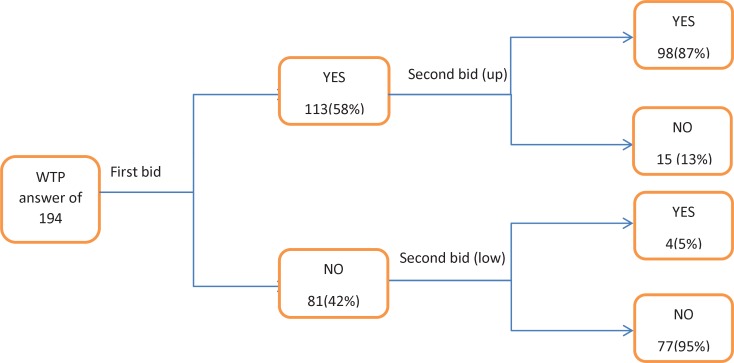
WTP responses to the first and second bid value

In this survey, among common forms of CVM, i.e. open ended, dichotomous choice, payment card and bidding game, we preferred using the dichotomous choice with a follow-up elicitation binary question- called, Double Bounded Dichotomous Choice (DBDC) - because of its efficiency, similarity to market and higher response rate (22, 23).

In the DBDC technique, an initial bid value was proposed to the respondents, if they accepted it, a higher bid was proposed, whereas if they had not accepted, the lower one would be proposed. To avoid a starting point bias, nine different starting point values were designed based on the information from the open-ended pretest pilot study and actual cost of 100 hospitalized patients at Modares hospital (Table 1). Then with an approximately equal distribution, each initial bid value was randomly allocated to one questionnaire.

In the present study, open-ended follow-up questions were asked to elicit more precious WTP. Additionally, for eliciting true WTP, we used ex-ante and ex-post approaches to minimize hypothetical bias of CVM studies. Formerly, as recommended by NOAA Panel, respondents were explicitly aware of their budget constraint and the financial consequent of extra payment on household budget. In latter, respondents were asked to determine financial resources of stated WTP amount (24). In this study, we used the ‘life-time’ model, that permits individuals to borrow/lend money for one-off payment (18). Financing options included saving, sales of assess, borrowing or reduction in household’s expenditure. 

*Data Analysis*

Excel 2010 and Stata 2013 were used for statistical analyses. 

There are two analytical approaches for driving WTP for QALY, namely aggregated and disaggregated. In the aggregated approach, which also called ratio of means, the mean value for QALY and WTP were estimated for all individual and then, the ratio of WTP for QALY were calculated by dividing the means. The disadvantage of this approach is, the inability to capture (take in to account) the preference of heterogeneity across respondents, while in the disaggregated approach, also known as the chained approach, first the WTP for QALY ratio was estimated directly for each respondent through elicited QALY gain and WTP at respondent’s level, then the mean of ratios were calculated to estimate WTP for QALY (25, 26). 

In this study, we preferred to employ the disaggregated approach to estimate the value of QALY through the following formula (27).

$\frac{\mathrm{WTP}}{\mathrm{QALY}}=\frac{\mathrm{willingness to pay amount}}{\sum_{t=1}^{\mathrm{life expectancy}}{\left(1-\mathrm{current utility}\right)*\left(1-\mathrm{discount rate}\right)}^{-(t-1)}}$

Where *r* is the discount rate and equals 5%, *t *represents the remaining life expectancy of each respondent. 

In this approach, we excluded respondents with full health state valuation from estimation, because of zero QALY at denominator which would result in an undefined value. Also, we employed the Heckman selection model -Two steps- (11, 28-29) to correct sample selection bias, while analyzing the effect of patients’ characteristics on WTP for QALY.

## Results

Patients’ characteristics are summarized in Table 3. A total of 194 patients referred from CCU, completed all assessments. The mean age was 57 years, 72% of patients were men and 78% of patients were head of household. Approximately, 14% had university education and 18% were illiterate. The majority of patients (82%) stated that, they were in household’s cost group of less than IRR 20,000,000 in a month. In the disease area, 57% of patients were admitted with coronary artery disease, 11% with heart failure, 8% with arrhythmia, and 42% of patients were hospitalized in the previous year.

The results of preference measurements are presented in Table 4. Based on the preference measures, the mean values for current health state, ranged from 0.59-0.71. As shown in the table, the highest utility value was produced by TTO technique in both the adjusted and 10-year model. It may be due to the existence of non-traders- respondents that did not trade any time to recover to full health. Therefore, the calculated utility value of them, was equal to one or full health state. In our study, the number of non-trades was 40 (21%) and 51 (26%) in TTO-adjusted and 10-year model respectively. Also, in EQ-5D and VAS assessment, 43(22%) and 4 (2%) of respondents had full health valuation, respectively. The mean value for WTP was estimated 300,000,000 Rials and 33 (17%) of individuals, stated to have zero value for WTP which was due to the inability to pay. Figure 1, presents the details of WTP responses.

Among the 194 respondents, a total of 113 patients had positive answer to the first proposed bid, i.e. they were willing to pay for the hypothetical treatment and recover to full health, while 81 respondents had a negative answer. For the second bid, among the 113 positive-answer respondents, only 15 individuals, rejected a higher bid value, and 4 individuals of 81 respondents with a negative response accepted the lower bid value (Figure1). Totally, 98 (51%) of respondents accepted the first and second bid values and 77 (40%) of them rejected 2 bid values among whom 33 out of 77 stated zero value for WTP mainly, because of the inability to pay. Interestingly, from the non-trader population in TTO-10 year, only 27% of individuals (14 0f 51) had zero WTP and the remaining were willing to pay.

The following tables (Tables 5 and 6 ) show the mean value of WTP for QALY for each health preference measures, the ratio between WTP for QALY, and GDP per capita and also effect of patients’ characteristics on the value of QALY. The average exchange rate from the Central Bank of Iran in 2014 was (IRR 28000 = USD 1). Also, Iran’s GDP per capita from the World Bank in 2014 was 4763 USD.

The mean discounted WTP for QALY in heart patients, ranged from IRR 76,168,710 to 100,767,560. This range was equal to 0.57-0.76 time of Iran’s local GDP per capita. 

In the disaggregated approach, all respondents with full health state valuation- in spite of positive WTP- were omitted from estimation because of zero QALY value at denominator. This omitting, produced a sample selection bias. Therefore, the Heckman selection model was used to correct selection bias and identify determinant factors-through analyzing data of censored and uncensored respondents-. The non-zero positive rho ratio of regression analysis in EQ-5D and TTO techniques indicated that censored data affected the value of QALY, while this ratio was close to 0 in VAS, 

which may be due to the existence of a few unobservable data. Therefore, we used multivariate regression analysis for predicting WTP/QALY calculated with VAS, which produced the same results. 

Our results showed that patients with a better health state and higher education, had higher WTP for QALY, in a significant positive manner (Table 6). Other significant variables associated with higher value of QALY include lower ages and more comorbidities (VAS, EQ_5D), marriage (TTO-adjusted) and higher household’s cost group as a proxy for income (VAS, EQ_5D and TTO 10-year).

## Discussion

Recently, the application of stated WTP to estimate monetary value of QALY, is increasing for policy implications and reimbursement decisions in spite of theoretical and analytical challenges surrounding it (26). In this study, we estimated the value of QALY using CVM through face-to-face interview with cardiovascular disease patients. There were several reasons behind this issue. Firstly, there was no explicit monetary value of QALY in Iran’s health system and consequently for interpreting CEA results and reimbursement decisions, WHO recommendation was used. Secondly, heart patients were targeted because CVDs are a serious growing health problem imposing enormous economic burden on the society and individuals and much effort and money were spent to manage and treat it. We interviewed hospitalized patients because they had been recently ill, and had a good perception of the illness severity and risks of death. Thus, we piloted this study in two heart hospitals to provide evidence for more informed decision making and also examine consistency of estimated value with WHO recommendation on using less than three GDP per capita as a threshold value for choosing cost-effective interventions (13). 

Our results indicate the mean discounted WTP for QALY, ranged from IRR 76,168,710 to 100,767,560. Although this value which is estimated by disaggregated approach and captures heterogeneity in health and WTP preferences of all respondents at individual level, it does not contain patients’ preference with zero QALY, even they had non–zero WTP to obtain health improvement. The problem of full health state valuation by patients may be related to preference measures, such as inability of generic measure (EQ-5D) to capture some conditions or aspects of specific diseases or its lower sensitivity rather than other instruments (30, 31), inappropriateness of TTO techniques especially 10-year time horizon for mild health states (32), patient’s adaptation with the disease (33), or unwilling to trade for some reasons such as hypothetical nature of the proposed treatment, non-severity of their current health state or family and children (the present study). If we compare the utility weight of non-traders in the 10-year model with the result from EQ-5D and VAS and even TTO-adjusted, the result indicated from 51 non-traders, a total of 19 and 4 individuals had full health state valuation in EQ-5D and VAS respectively. 

Others, however, valuated their health less than full health. This difference in health state valuation result, indicated that for non-trader patients, the QALY gain may be small, but it necessarily could not be a zero. For as much as these respondents constituting 20-25% of the sample, for considering them in value estimation, two options were available: employing the aggregated approach irrespective of patients’ preferences at individual level or using disaggregated approach while assigning an arbitrary utility value to these respondents. For the latter option, in our analysis, we derived mean utility value of TTO- traders for different age groups and assign theses values to non-traders of same age-group with the assumption that similarity existed within respondents in each age- group. In the aggregated analysis, the estimated mean value of QALY are IRR 63,181,750 (EQ-5D), IRR 67,312,450 (VAS), IRR 86,649,180 (TTO-adjusted) and IRR 87,833,700 (TTO 10-years). The mean value of QALY in the second option equals IRR 55,547,340 (EQ-5D), IRR 83,024,350 (TTO-10 year), IRR 86,158,030 (TTO- adjusted), and IRR 90,537,440 (VAS). In all approaches, the estimated value of QALY was less than one GDP per capita. This is consistent with one of our other studies that estimates the value of QALY in diabetic patients in (in press). The results are also in line with Nimdet’ systematic review indicating that among 167 estimated WTP for QALY from 14 studies, 76% were below one GDP per capita (34). Therefore, in spite of the WHO recommendation, indicating that less than 3 GDP per capita as a threshold value should be used, ICER value of more than one is not advocated for our health system.

Finally, regarding to this issue that, the disaggregated approach is a preferable method (25, 35), we report the range of IRR 76,168,710 to 100,767,560 as a monetary value of QALY from heart a patient’s perspective. However, we could not claim with certainty that our value is a definite preference of all heart patients because it is a pilot study with a limited sample size from public-funded hospitals that estimated WTP for QALY through quality of life improvement. Future studies that consider more representative sample for extending or saving life aspects are suggested (36,37). 

## Conclusion

The WTP for QALY could be estimated from different analytical approaches. In this study, although the disaggregated approach produced higher values, but all estimated values were less than one GDP per capita. Therefore, for policy implication and using monetary value of QALY as a CE threshold value for reimbursement decision, our result did not advocate the range (1-3 time) of GDP per capita (more than one GDP per capita) that is recommended by WHO. Also, because the disaggregated approach is a preferable method for estimating the monetary value of QALY and thanks to capture heterogeneity in individual preferences, the main challenge concerned individuals with zero QALY gain; therefore, more scrutiny is suggested on how to deal with respondents with full health valuation and what arbitrary value they could include in analysis. 
